# Analysis of primary care of victims of interpersonal and self inflicted violence during the COVID-19 pandemic

**DOI:** 10.1590/0100-6991e-20233423-en

**Published:** 2023-03-31

**Authors:** HELOÍSA MORO TEIXEIRA, ANGEL ADRIANY DA-SILVA, ANNE KAROLINE CARDOZO DA-ROCHA, MARIANA ROTHERMEL VALDERRAMA, RAFAELLA STRADIOTTO BERNARDELLI, VITÓRIA WISNIEVSKI MARUCCO SILVA, LUIZ CARLOS VON BAHTEN

**Affiliations:** 1 - Hospital Universitário Cajuru, Liga Acadêmica do Trauma (LATHUC) - Curitiba - PR - Brasil; 2 - Pontifícia Universidade Católica do Paraná, Escola de Medicina e Ciências da Vida - Departamento de Bioestatística - Curitiba - PR - Brasil; 3 - Universidade Federal do Paraná, Departamento de Clínica Cirúrgica - Curitiba - PR - Brasil; 4 - Pontifícia Universidade Católica do Paraná, Escola de Medicina e Ciências da Vida - Departamento de Clínica Cirúrgica - Curitiba - PR - Brasil

**Keywords:** Epidemiology, COVID-19, Aggression, Traumatology, Wounds and Injuries, Agressão, Ferimentos e Lesões, Epidemiologia, Traumatologia, COVID-19

## Abstract

**Objectives:**

to analyze the epidemiological profile of aggression victims admitted at the emergency room on a trauma hospital during the COVID-19 pandemic, to compare these data in different restriction periods and with prepandemic data from the same service.

**Methods:**

cross-sectional study with probabilistic sampling using medical records of patients who were victims of aggression admitted at the hospital between June 2020 and May 2021. In addition to the epidemiological variables, other variables collected were the current restriction level, mechanism of aggression, resulting injuries and the Revised Trauma Score (RTS). The data was compared between the three restriction levels and the proportion of attendances during the study period was compared with the pre-pandemic study (December 2016 to February 2018).

**Results:**

the average age was 35.5 years, 86.1% of the patients were male and 61.6% of the attendances were due to blunt injury. The highest average of attendances per day occurred during the “yellow” restriction level (2.9), however there was no significant difference when comparing the restriction periods two by two. There was also no significant difference either in the analysis of the standardized residuals of the proportions of aggressions or the mechanism of aggression in the pre-pandemic and pandemic periods.

**Conclusions:**

there was a predominance of attendances due to blunt trauma and in young male patients. There was no significant difference between the average daily attendance for aggression during the three restriction levels and between the proportion of attendances in the pre-pandemic and pandemic period.

## RESUMO

**Objetivo:**

analisar o perfil epidemiológico dos pacientes vítimas de agressão admitidos na sala de emergência de um hospital de trauma durante a pandemia da COVID-19, comparar tais dados entre os diferentes períodos de restrição e com os dados de um estudo pré-pandêmico do mesmo hospital.

**Métodos:**

estudo transversal com amostragem probabilística utilizando prontuários de pacientes vítimas de agressão admitidos no referido hospital entre junho de 2020 e maio de 2021. Além das variáveis epidemiológicas, coletou-se o nível de restrição vigente, o mecanismo de agressão, as lesões resultantes e o Revised Trauma Score (RTS). Os dados foram comparados entre os três níveis e a proporção de atendimentos no período do estudo foi comparada com a do estudo pré-pandêmico (dezembro de 2016 a fevereiro de 2018).

**Resultados:**

a média etária foi de 35,5 anos, 86,1% dos pacientes eram do sexo masculino. e 61,6% dos atendimentos corresponderam a agressão física por ferimento contuso. A maior média de atendimentos por dia ocorreu durante o nível amarelo (2,9), contudo não houve diferença significativa nos atendimentos quando comparados os períodos de restrição dois a dois. Também não houve diferença significativa tanto na análise dos resíduos padronizados das proporções de agressões quanto em cada mecanismo de agressão nos períodos pré-pandêmico e pandêmico.

**Conclusões:**

houve predominância de atendimentos por agressão por trauma contuso e em pacientes jovens do sexo masculino. Não houve diferença significativa entre a média diária de atendimentos por agressão durante os três níveis de restrição implementados e entre a proporção de atendimentos do período pré-pandemico e pandêmico.

## INTRODUCTION

The World Health Organization (WHO) defines violence as the intentional use of physical force or power against a person, group or community, resulting in injury, death, psychological harm, maldevelopment, or deprivation. Violence can be interpersonal, when it occurs between family members, partners, friends, acquaintances, or strangers, or self-inflicted, which includes suicidal behavior and self-mutilation^1^.

More than 1.3 million people die annually as a result of violence, which is equivalent to 2.5% of global mortality. Among the population aged 15-44 years, violence represents the fourth cause of death in the world^1^. In 2019, Brazil recorded 45,503 homicides, a rate of 21.7 deaths per 100,000 inhabitants, the lowest number since 1995. However, the Atlas of Violence highlights that there has been a decrease in the quality of official records^2^. In this context, the importance of the continuous survey of epidemiological data on this event and the resulting injuries is evident, to identify changes in morbidity and mortality, optimize medical care, and motivate the implementation of preventive public policies^3^.

In 2020, another disease became evident and was declared a pandemic. The disease caused by the 2019 Coronavirus (COVID-19), with the first case reported in 2019, is highly transmissible by particles in the upper airways. Although the majority of those infected are asymptomatic, which increases the number of cases, it can progress to respiratory failure, requiring hospital support^4^ ^,^ ^5^. Thus, to contain its spread and reduce the burden on health systems, several governments have adopted social distancing policies and protocols of non-pharmacological measures at different restriction levels^6^. In June 2020, the Municipality of Curitiba (Brazil) implemented the Sanitary and Social Responsibility Protocol, which presented three restriction levels on the movement of people according to the COVID-19 transmission risk. Such levels were presented to the population in the form of a color scheme, in which the first level, called yellow, defined an alert situation, encouraging health and social responsibility measures. The second, orange level, represented moderate risk and instituted some restrictions on the functioning of services, shops, and areas that favor agglomeration. Finally, the high-risk level, called red, restricted the movement of people and allowed only essential services to operate^7^.

It was anticipated that the restrictions would generate an overall decrease in the number of trauma cases by reducing the movement of people, with a lower risk of traffic accidents or interpersonal violence^8^. Thus, the objective of this study was to evaluate the epidemiological profile of victims of aggression admitted to the emergency room of a reference hospital in the city of Curitiba-PR during the COVID-19 pandemic, to assess whether there was a difference in attendance between the restriction levels implemented in this period and to compare the results with those of the period prior to the pandemic.

## METHODS

This is a cross-sectional study with probabilistic sampling of medical records of patients admitted to a University Hospital in Curitiba-PR between the period from June 2020 to May 2021. Patients who were victims of aggression aged 18 years or over, admitted via the emergency room were included by direct search or brought by medical rescue service. We excluded incomplete medical records or those of patients who died on arrival at the hospital.

The computation of the sample size was performed based on data from the study in the period prior to the pandemic at the same institution (December 2016 to February 2018)^9^ and the initial sample of 100 successive consultations that occurred during the pandemic. In order to detect a significant difference between the distributions on the trauma mechanism classifications (assaults, traffic accidents, and falls), when comparing the pre-pandemic and during the pandemic periods, a total of 833 patients would be necessary, considering the significance level of 5% and test power of 80%. Sampling of medical records occurred using the GraphPad software. On alternate days, one fifth of the medical records were drawn, following the inclusion criteria.

The variables collected were age, sex, type of sustained aggression, day of the week on which it occurred, current restrictive level (yellow, orange, or red), whether it was a holiday, and which transport service took the patient to the hospital. Variables of initial in-hospital care were Glasgow Coma Scale (GCS), systolic blood pressure (SBP), and respiratory rate (RR) for calculation of the Revised Trauma Score (RTS), whether the massive transfusion protocol was activated, injured body regions, presence of trauma to the extremities, exposed fracture, traumatic brain injury (TBI), need of hospitalization, alcohol and/or drug consumption prior to the event, and suicide attempt (self-harm).

Among the medical rescue services, there is the Integrated Trauma Care Service (SIATE), which was created in 1990 in Curitiba, is integrated with the Fire Department, and aids trauma victims. There is also the Mobile Emergency Care Service (SAMU), which was created in 1995 and exists throughout Brazil, being responsible for attending to all types of medical emergencies, including trauma^9^. The RTS is a physiological score that allows assessing the morbidity and mortality of polytraumatized patients. Its values vary between 0 and 8, allowing for fractions, and the higher the final value, the better the patient’s prognosis^10^.

The collected data were recorded in a Microsoft Excel^®^ spreadsheet and analyzed with the IBM SPSS Statistics v.20.0 software, Armonk, NY, IBM Corp. Age results were described by mean, standard deviation, minimum and maximum, and categorical variables, by frequency and percentage. We used the Kruskal-Wallis non-parametric test to compare the GCS and RTS between the three groups established by the restriction levels. We used the chi-square test to analyze the associations of categorical variables related to aggression with the three restriction levels. The periods defined by the three levels were compared two by two in relation to the occurrences of assistance due to aggression. The Chi-square test was used to compare the proportion of visits due to aggression and others (traffic accidents and falls) performed during the pandemic period with pre-pandemic visits in the same hospital^9^. We also compared the proportion of aggression mechanisms (physical assault, gunshot wounds, stab wounds) from the pandemic period with the pre-pandemic one^9^. Values of p<0.05 indicated statistical significance. For the analyzes that showed statistical significance in the chi-square test, we analyzed the residuals, considering that there is an association between the variables in the cells that have adjusted standardized residuals value greater than 1.96. No strategies were adopted to correct missing data.

The project was approved by the Ethics in Research Committee and has the Certificate of Presentation of Ethical Appreciation (CAAE) number 40014320.2.0000.0020, and opinion number 4573831.

## RESULTS

We included 172 patients, whose mean age was 35.5 years, with a standard deviation of 11.5 (18-72 years). The predominant age group was 18 to 29 years old (37.2%) and 86.1% of the patients were male (Table 1).

**Table 1:** Epidemiology of aggressions during the COVID-19 pandemic.

Variable	Classification	n=172 n(%)
Age (years)	18 a 29	64 (37.2%)
	30 a 39	46 (26.7%)
	40 a 49	43 (25%)
	50 a 59	14 (8.1%)
	60 a 69	2 (1.2%)
	≥70	3 (1.7%)
Sex	Female	24 (13.9%)
	Male	148 (86.1%)
Mechanism of Aggression	Physical aggression	106 (61.6%)
	Stab Wound	34 (19.8%)
	Gunshot wound	25 (14.5%)
	Self-inflicted	7 (4.1%)
Resulting injuries (anatomical region)	Face	42 (24.4%)
	Head neck	44 (25.6%)
	Upper limbs	42 (24.4%)
	Chest	28 (16.3%)
	Abdomen	17 (9.9%)
	Back	12 (7%)
	Pelvis/hip	12 (7%)
	Lower members	26 (15.1%)
	External surfaces	56 (32.6%)
Variable	Classification	n=172 n(%)
Alcohol consumption	Not	143 (83.1%)
	Yes	29 (16.9%)
Consumption of other drugs	Not	134 (77.9%)
	Yes	38 (22.1%)
Day of the week	Monday	23 (13.4%)
	Tuesday	20 (11.6%)
	Wednesday	23 (13.4%)
	Thursday	23 (13.4%)
	Friday	26 (15.1%)
	Saturday	29 (16.9%)
	Sunday	28 (16.3%)
Transport	SIATE	93 (54.1%)
	SAMU	73 (42.4%)
	Direct search	3 (1.7%)
	Helicopter	2 (1.2%)
	Highway rescue service	1 (0.6%)

As for the days of the week, Saturday and Sunday had the highest number of visits (Table 1) and eight patients were seen on holidays. Most were taken to the hospital via SIATE (54.1%) and SAMU (42.4%) (Table 1).

Regarding the aggression mechanism, most patients suffered physical aggression with blunt injury (61.6%) (Table 1). Of the seven patients attended for self-harm, three were due to exogenous intoxication (1.7%), two due to self-harm (1.2%), and two due to hanging (1.2%). Consumption of alcohol and other drugs was reported by 16.9% and 22.1% of patients, respectively (Table 1).

The average GCS was 14.25 and the median, 15 (3-15). The RTS mean and median were 7.65 and 7.8, respectively, the highest value found being 7.8 and the lowest, 2.3.

As for the body region, the most injured were external surface due to blunt injury, head and neck, face, upper limbs, chest, and lower limbs (Table 1). In addition, 15.1% (n=26) suffered trauma to the extremities, 23.07% (n=6) of these with open fractures, and 9.3% (n=16) suffered TBI, the mean GCS being 14.25. Of the total, 34.3% (n=59) required hospitalization to resolve the clinical condition and only 1.2% needed massive transfusion.

Regarding the restriction levels implemented, the highest average of calls per day occurred during the yellow level, 2.9. However, when comparing the periods two by two, there was no significant difference between the levels yellow and orange (p=0.134), yellow and red (p=0.308), or orange and red (p=0.643). There was a significant difference in reported alcohol consumption between the three restriction levels (p<0.001), with a significantly higher proportion in the red one. As for injuries, there was a significant difference between the groups of patients who were treated during each of the restriction levels regarding the occurrence of injuries to the face (p=0.011), to external surfaces (p<0.001), and to TBI (p=0.034). There was a proportionally greater occurrence of injuries to the face during the yellow level, injuries to external surfaces also during the yellow level, and TBIs during the red level (Table 2).

**Table 2:** Categorical variables related to aggression according to restriction levels.

		Restriction Levels			
Variable	Classification	Yellow (n=57)	Orange (n=101)	Red (n=14)	p*
Sex	Female	5 (8.8%)	16 (15.8%)	3 (21.4%)	
	Male	52 (91.2%)	85 (84.2%)	11 (78.6%)	0.329
Mechanism of Aggression	Physical aggression	35 (61.4%)	62 (61.4%)	9 (64.3%)	0.151§
	Stab wound	17 (29.8%)	16 (15.8%)	1 (7.1%)	
	Gunshot wound	5 (8.8%)	17 (16.8%)	3 (21.4%)	
	Suicide attempt	0 (0%)	6 (5.9%)	1 (7.1%)	
Reason for Aggression	Discussion	4 (7%)	3 (3%)	1 (7.1%)	
	Robbery	53 (93%)	98 (97%)	13 (92.9%)	0.459
Face injury	No	51 (89.5%) [3.0]^#^	70 (69.3%) [-2.3]^#^	9 (64.3%) [-1.0]^#^	0.011
	Yes	6 (10.5%) [-3.0]^#^	31 (30.7%) [2.3]^#^	5 (35.7%) [1.0]^#^	
Head and neck injury	No	46 (80.7%)	75 (74.3%)	7 (50%)	0.062
	Yes	11 (19.3%)	26 (25.7%)	7 (50%)	
Upper limb injury	No	47 (82.5%)	77 (76.2%)	6 (42.9%)	0.008
	Yes	10 (17.5%)	24 (23.8%)	8 (57.1%)	
Chest injury	No	47 (82.5%)	87 (86.1%)	10 (71.4%)	0.358
	Yes	10 (17.5%)	14 (13.9%)	4 (28.6%)	
Abdomen injury	No	51 (89.5%)	93 (92.1%)	11 (78.6%)	0.278
	Yes	6 (10.5%)	8 (7.9%)	3 (21.4%)	
Back injury	No	55 (96.5%)	92 (91.1%)	13 (92.9%)	0.441
	Yes	2 (3.5%)	9 (8.9%)	1 (7.1%)	
Pelvis/hip injury	No	55 (96.5%)	92 (91.1%)	13 (92.9%)	0.441
	Yes	2 (3.5%)	9 (8.9%)	1 (7.1%)	
Lower limb injury	No	52 (91.2%)	84 (83.2%)	10 (71.4%)	0.136
	Yes	5 (8.8%)	17 (16.8%)	4 (28.6%)	
Injury to external surfaces	No	28 (49.1%) [-3.6]^#^	74 (73.3%) [1.9]^#^	14 (100%) [2.7]^#^	<0.001
	Yes	29 (50.9%) [3.6]^#^	27 (26.7%) [-1.9]^#^	0 (0%) [-2.7]^#^	
Orthopedic Trauma	No	51 (89.5%)	84 (83.2%)	11 (78.6%)	0.449
	Yes	6 (10.5%)	17 (16.8%)	3 (21.4%)	
Exposed fracture	No	56 (98.2%)	98 (97%)	12 (85.7%)	-
	Yes	1 (1.8%)	3 (3%)	2 (14.3%)	
TBI	No	53 (93%) [0.7]^#^	93 (92.1%) [0.7]^#^	10 (71.4%) [-2.6]^#^	0.034
	Yes	4 (7%) [-0.7]^#^	8 (7.9%) [-0.7]^#^	4 (28.6%) [2.6]^#^	
Need of hospitalization	No	39 (68.4%)	66 (65.3%)	8 (57.1%)	0.723
	Yes	18 (31.6%)	35 (34.7%)	6 (42.9%)	
Alcohol consumption	No	50 (87.7%) [1.1]^#^	87 (86.1%) [1.3]^#^	6 (42.9%) [-4.2]^#^	<0.001
	Yes	7 (12.3%) [-1.1]^#^	14 (13.9%) [-1.3]^#^	8 (57.1%) [4.2]^#^	
Consumption of other drugs	No	45 (78.9%)	80 (79.2%)	9 (64.3%)	0.439
	Yes	12 (21.1%)	21 (20.8%)	5 (35.7%)	

Result described in frequency (percentage). *Chi-square test, p<0.05. ^§^The comparison was made between the physical aggression, stab wounds, and gunshot wounds groups. Suicide attempts were excluded from the analysis given the low frequency of cases. ^#^Adjusted standardized residuals that, for each cell, result from (observed frequency - expected frequency)^2^ ÷ expected frequency. Cells with values greater than 2 indicate significant association/difference between variables. Positive residuals indicate a direct relationship, while negative ones indicate an inverse relationship. Residuals were presented in cases of significant association by the chi-square test

As for RTS, there was no significant difference between the restriction levels, and the mean with the highest value was during the yellow one (7.8) (Table 3).

**Table 3:** RTS scores according to each restriction level.

Restriction level	RTS score					p*
	n	Average	Median	Minimum	Maximum	
Yellow	57	7.8	7.8	5.0	7.8	
Orange	101	7.6	7.8	2.3	7.8	0.098
Red	14	7.6	7.8	6.0	7.8	

*Kruskal-Wallis non-parametric test, p<0.05.

The proportions of trauma mechanisms traffic accidents, assaults, and falls were significantly different between the pre-pandemic and during the pandemic periods (p=0.031). In the analysis of standardized residuals, there was a significantly higher proportion of assistance for car accidents in the period prior to the pandemic and a significantly greater proportion of falls during the pandemic. However, there was no significant difference in the proportions of aggressions in the two periods (Table 4).

**Table 4:** Proportions of visits due to the three trauma mechanisms between the pre-pandemic ^9^ and pandemic periods.

Trauma mechanism	Pre-pandemic^9^ (2016-2018)	COVID-19 pandemic (2020-2021)	p*
	n (%) - [Residual^#^]	n (%) - [Residual^#^]	
Traffic-accidents	658 (53.1%) - [2.49]	426 (47.7%) - [-2.49]	
Aggressions	229 (18.5%) - [-0.44]	172 (19.2%) - [0.44]	0.031
Falls	352 (28.4%) - [-2.33]	296 (33.1%) - [2.33]	
Total	1,239 (100%)	894 (100%)	

*Chi-square test significance, p<0.05. ^#^Adjusted standardized residuals that, for each cell, results from (observed frequency - expected frequency) ^2^ ÷ expected frequency. Cells with values greater than 2 indicate significant association/difference between variables. Positive residues indicate a direct relationship, while negative ones indicate an inverse relationship.

[Table t5]

**Table 5 t5:** Proportions of the three aggression mechanisms between the pre-pandemic ^9^ and pandemic periods.

Mechanism of Aggression	Pre-pandemic^9^ (2016-2018)	COVID-19 pandemic (2020-2021)	p*
	n (%) - [Residual^#^]	n (%) - [Residual^#^]	
Physical aggression	152 (66.4%)	106 (64.2%)	0.653
Stab Wound	39 (17.0%)	34 (20.6%)	
Gunshot wound	38 (16.6%)	25 (15.2%)	
Total	229 (100%)	165 (100%)	

*Chi-square test significance, p<0.05.

When only consultations due to aggression were compared, there was also no evidence of a significant difference in the proportion of aggression mechanisms (physical aggression, stab wounds, or gunshot wounds) assisted in the pre-pandemic period and during the pandemic (p=0.653). The self-harm mechanism was not considered in this comparison due to the lack of such information in the pre-pandemic period.

## DISCUSSION

The results of this study show an epidemiological profile composed of young male victims of aggression due to blunt trauma. This data is similar to that found in the pre-pandemic study carried out at the same institution, in which the incidence of males was above 80%^9^. Another study carried out in the United Kingdom showed that most patients admitted due to penetrating trauma between 2020 and 2021 were men, with a mean age of 28.2 years^11^. Due to social construction, men are more exposed to risk situations and physical violence, which could explain the results^9^ ^,^ ^12^.

A higher volume of interpersonal violence consultations on weekends (Saturdays and Sundays) was also seen in the present study. In contrast, a study carried out in Qatar with trauma patients in the pre-pandemic period showed no significant difference in admissions on weekdays and weekends^11^. As for the medical rescue service, there was a predominance of SIATE in pre-hospital care, followed by SAMU, similar to pre-pandemic results (55.5% and 44.5%, respectively)^9^. The small difference between these rescue systems found in our study may demonstrate the coexistence and complementarity of both in the health service.

Regarding the aggression mechanism, there was a predominance of aggression due to blunt trauma, which agrees with other studies. In research conducted in Southern California during the pandemic, of the 1,229 attended for aggression, 36.4% (n=448) were for physical aggression, 24.4% (n=300) for stab wounds, 29% (n=356) due to gunshot wounds, and 10.2% (n=125) due to suicide attempts^13^. When comparing the results of this study between the restriction levels and with the pre-pandemic period, there was no significant difference in the proportion of attendances and each aggression mechanism, nor in the incidence of aggression between the three levels, similar to two North American studies^14^ ^,^ ^15^. There was no significant change in the occurrences of penetrating trauma^14^, nor in care for injuries involving physical aggression or with a blunt object and for penetrating injuries in relation to the most restrictive period (“Stay-at-home”)^15^. Restrictive measures reduced the movement of people, and one would expect a reduction in this trauma mechanism or change in its profile. However, exposure to stressors such as the need for seclusion, anxiety, fear of the disease, and socioeconomic factors affected the population’s mental health^14^ ^,^ ^16^, which may have contributed to the maintenance of the number of attendances due to aggression in the pandemic.

As for the body region, in addition to the predominance of superficial injuries, head and neck, face, and limbs, there was involvement of the thorax, which is similar to another study with a predominance of physical aggression^17^. However, the median RTS of this study was 7.8 and there was no significant difference in RTS scores between the analyzed restriction periods, which agrees with a South Korean study during the pandemic^18^. Patients with blunt or penetrating trauma in the thoracic and abdominal region can maintain their level of consciousness during initial care, unlike TBI victims^10^. Since the highest weight in RTS is the GCS score, the results found in both studies are justified.

Regarding the consumption of alcoholic beverages and the use of illicit drugs, our results are similar to those of Freitas et al. (2017), in which the use of illicit drugs occurred in 65.31% of the young people interviewed, and alcohol consumption in 31.2%^19^. Interpersonal violence related to the consumption of alcohol and drugs usually involves men and occurs in public environments, such as bars and streets. Its abusive use has been associated with a higher occurrence of injuries resulting from violence, as it is a potentiating element of aggressive acts^17^.

## CONCLUSIONS

The epidemiological profile of victims of aggression treated during the COVID-19 pandemic was young men who were victims of interpersonal violence due to blunt injury. There was no significant difference between the daily average of attendances due to aggression during the three restriction levels implemented and in the comparison between the proportions of attendances due to aggression and each of its mechanisms in the pandemic period with the pre-pandemic one.
